# The Effects of Intrapersonal Anger and Its Regulation in Economic Bargaining

**DOI:** 10.1371/journal.pone.0051595

**Published:** 2012-12-26

**Authors:** Emma C. Fabiansson, Thomas F. Denson

**Affiliations:** School of Psychology, University of New South Wales, Sydney, Australia; George Mason University/Krasnow Institute for Advanced Study, United States of America

## Abstract

Anger is a common cause of strained negotiations. This research investigated the effects of experiencing anger (Experiment 1) and regulating anger (Experiment 2) on ultimatum bargaining. Experiment 1 showed that relative to a control condition, angered participants proposed less fair offers and rejected more offers when bargaining with the person who angered them. Experiment 2 replicated Experiment 1, and additionally showed that regulating anger via reappraisal and distraction both reduced anger. However, only reappraisal effectively reduced anger for the duration of the negotiation. Participants who reappraised proposed fairer offers than those in the distraction condition, but did not differ in offers accepted. This research may have implications for what emotion regulation strategy should be employed in economic bargaining. However, future research is required to determine the most effective timing and components of reappraisal for promoting beneficial outcomes in bargaining contexts.

## Introduction

Negotiations and bargaining often break down due to feelings of anger. Anger can cloud judgment and make it difficult to act rationally, which may cause poor economic outcomes and relationship conflict. Understanding how to regulate the experience of anger in negotiations has received little attention to date. To address this problem, we investigated the effects of experiencing and regulating anger in the latter stages of the negotiation process, using an economic bargaining game. Specifically, we experimentally tested and confirmed the hypothesis that being angered adversely affects bargaining outcomes (Experiment 1). We then show that one emotion regulation strategy in particular - cognitive reappraisal - can reduce anger and increase fairness in economic bargaining situations (Experiment 2).

### Intrapersonal Anger

Van Kleef and colleagues distinguish between *inter*personal and *intra*personal anger [1]. *Interpersonal anger* refers to the effect of one person's display of anger on another individual. Expressing anger tends to improve financial outcomes for the expresser. Anger displays convey toughness and that the person expressing anger has high bargaining limits [1], [2]. Multiple studies have shown that angry opponents elicit greater concessions than happy opponents in both negotiations and ultimatum bargaining [1], [3]–[5].

By contrast, *intrapersonal anger* refers to feeling angry. Intrapersonal anger can arise when one's goals have been blocked and the blockage is attributed to a blameworthy person or object [6], [7]. Anger is a negatively valenced, high arousal, approach-related emotion [6], [8] that has specific effects on our physiology and cognition. Anger is energizing, and can increase reliance on heuristic decision-making [9]–[11]. This kind of non-analytic processing may be most likely to occur when anger is characterized by high certainty and strong arousal [12]. Furthermore, anger causes people to want to change their current situation via goal restoration or inflicting harm [7], [8].

Intrapersonal anger can be detrimental to negotiations because it facilitates competitive and retaliatory behavior [13]–[15]. Anger involves appraisals of certainty and control that make negotiators feel powerful [16]. Angry negotiators have a high concern for themselves, with little consideration of others [15]. Bargaining individuals who are focused on their own interests are unlikely to understand what their counterpart values or realize opportunities for mutual gain [14]. In contrast to other emotional states including pride-achievement, gratitude, and guilt-shame, research shows that intrapersonal anger increases competitive and dominating behavior [17]. In sum, angry individuals may be quick to act, aggressive, try to assert dominance, and show little consideration of others during negotiations and bargaining. Collectively these studies illustrate that intrapersonal anger may have detrimental financial effects.

### The Ultimatum Game and Aggressive Retaliation

We examined bargaining behavior within the context of the Ultimatum Game. The Ultimatum Game is a simple yet powerful research tool based on a “take it or leave it” principle [18]. The game involves two roles: the proposer and the responder. The proposer divides the money. The responder chooses whether to accept or reject the offer. If the responder accepts the offer, the money is divided accordingly. Rejecting the offer comes at a personal cost, as both parties receive no money. Thus, the responder can punish the proposer for receiving an unsatisfactory offer by rejecting it. This paradigm models the final stages of the negotiation process and has been extensively used in prior research [13], [19], [20]. The Ultimatum Game mirrors real life negotiations as ultimatum offers are common occurrences. Real world examples include a recruiter giving a final salary offer to the best candidate or a car salesperson making their final offer when selling a car. Such instances can be modeled with the Ultimatum Game.

Prior research shows that receiving unfair offers can induce anger in the Ultimatum Game [13]. One might assume that responders should accept any offer regardless of the size of the offer as receiving some money is objectively better than receiving none. However, offers of less than 20% of the total amount are typically rejected [21]. Pillutla and Murnighan [13] showed that when participants were able to evaluate the fairness of the offer, low offers were rejected to punish the proposer for unfair treatment. Moreover, self-reported anger was a stronger determinant of whether participants rejected offers than the perceived fairness of the offers. Similarly, studies using other bargaining tasks have also shown that physiological arousal and self-reported anger are associated with punishment decisions [22].

In the present study, we examined how being angered by one's bargaining partner prior to bargaining might influence behavior in the Ultimatum Game. This is of theoretical and practical interest, as people must sometimes bargain with a disliked partner. Moreover, accepting an unfair offer in the Ultimatum Game requires one to regulate anger and resist impulsively punishing the proposer [23]. This seems particularly difficult to do when angry. Thus, we expected that angry participants would engage in retaliation by rejecting offers from the provoking person and proposing unfair offers to that person.

### Emotion Regulation: Reappraisal and Distraction

Emotion regulation broadly refers to how people control their experience and expression of emotions by selecting which emotions they experience, how they experience these emotions, and when they experience them [24]. Only a handful of studies have investigated how to regulate anger in negotiations. Previous suggestions for regulating anger in negotiations lack empirical testing. For instance, some have suggested venting anger, leaving the negotiation room, and exercising before or after the negotiation [25], [26]. However, venting anger increases or maintains rather than decreases anger [27], [28]; leaving the room is not always possible or desirable, and exercise increases arousal which can increase aggression [29].

There is some initial evidence from neuroeconomics that regulating anger may be effective in reducing retaliation in the Ultimatum Game. Receiving unfair ultimatum offers activates regions associated with anger and other negative gut-level emotional reactions such as the anterior insula [20], [23], [30]. In terms of behavior, people who are better able to down-regulate the negative affect associated with unfair offers are less likely to act on their emotional impulses and reject unfair offers. The neural evidence supports this notion. For instance, in Tabibnia et al.'s [23] study, accepting unfair offers was associated with decreased anterior insula activity and greater right ventrolateral prefrontal cortex activation. This latter region is implicated in emotion regulation and inhibition [31], [32]. In another study, individuals with damage to the ventrolateral prefrontal cortex were more likely to reject offers than healthy controls [33]. Taken together, these findings suggest that regulating negative affect may be important for accepting unfair but financially beneficial offers.

How might individuals regulate anger when confronted with an unfair offer? Independent of negotiation and bargaining contexts, two emotion regulation strategies have proven effective in reducing anger: cognitive reappraisal and distraction.

#### Cognitive Reappraisal

Cognitive reappraisal involves reinterpreting an anger-eliciting event into neutral, less emotional terms by considering the event from a non-personal, objective perspective [24]. Reappraisal may also include consideration of any positive aspects of the event, such as lessons learned.

Cognitive reappraisal is a very effective strategy for reducing anger and adverse cardiovascular responses to interpersonal hostility [34]–[36]. However, the timing of reappraisal is important. According the Gross' process model of emotion regulation, reappraisal should take place before the full onset of an emotion if it is to effectively change the experience [37]. As an illustration, reappraisal may occur when an employee notices that his or her supervisor is having a bad day and is subsequently insulted by their supervisor. The employee who reappraises may experience some initial anger, but subsequently lower their angry feelings by not interpreting the provocation as a personal and intentional attack.

#### Distraction

Distraction involves directing attention away from the anger-provoking event to unrelated neutral or positive stimuli [38]. For example, in the workplace, distraction may include directing anger away from an anger-inducing incident by thinking about the layout of the office. Relative to thinking about an anger-inducing event, distraction following anger provocation also reduces intrapersonal anger [28], [39], [40].

At first glance, these findings suggest that distraction may be quite helpful for regulating anger in bargaining contexts. However, we suggest that one key difference between distraction and reappraisal is that reappraisal entails cognitively processing the event whereas distraction does not. Distraction may be beneficial in promoting quick recovery because it does not allow individuals the opportunity to ruminate [41], [42]. However, some form of thinking about the anger provocation may be required in order to effectively reduce emotional and physiological responses induced by the event. Thus, distraction may produce an immediate reduction in anger, but when one must subsequently bargain with a disliked individual, the unresolved anger may flare up again. By comparing reappraisal to distraction across time, we tested this possibility.

### The Present Research

To our knowledge, only one study has examined how emotion regulation affects bargaining behavior. Wang et al. [43] (Study 3A) applied the notion of “counting to 10” when angered to ultimatum and third-party punishment contexts. Undergraduates received anger-inducing unfair offers in the Ultimatum Game. Prior to deciding whether to accept or reject, there was a 30 sec or 2.5 min time delay. During this delay, participants wrote about a trip to the grocery store (i.e., distraction), what they were feeling (i.e., a proxy for rumination), or what they were thinking (i.e., a proxy for reappraisal). No differences in punishment were found between the three emotion regulation conditions following the 30 sec delay. However, following the 2.5 min delay period, participants who wrote about what they felt punished more than participants who wrote about what they thought and those in the distraction condition. The latter two conditions did not significantly differ in punishment.

The research we present here acknowledges and extends Wang et al.'s work in several ways. First, because real world bargaining sometimes occurs between individuals who have a history of hostility, we manipulated this feature of the bargaining context. Second, Wang et al. acknowledge that in their study reappraisal was induced very generally (i.e., write about what you are thinking). We used a more standard reappraisal induction, which included being directed to focus on the event objectively or from the perspective of a third person. They also asked participants to write about what they were thinking *after* receiving the anger-inducing unfair offer rather than prior to the anger induction. Thus, we included a directed antecedent-focused reappraisal procedure based on prior work within the context of the process model of emotion regulation [37], [44]–[46]. According to the process model, reappraisal should be most effective in lowering anger when initiated prior to a full emotional response [44], [47]. Third, reappraisal and distraction may vary in terms of how effective they are in reducing anger in the shorter and longer-term. Thus, we examined the time course of anger during the experiment. Fourth, we allocated participants to play both the role of the proposer as well as the responder. Including the proposer allowed us to examine the possibility that reappraisal and distraction would influence prosocial behavior, which was operationalized as making fairer offers.

Across two experiments we tested the effects of intrapersonal anger on economic bargaining. In Experiment 1, we examined whether intrapersonal anger would lead to poor bargaining outcomes in two tasks. We expected that provoked participants would propose less fair ultimatum offers to the provoking counterpart relative to unprovoked participants. We also expected that provoked participants would accept fewer offers from the anger-provoking counterpart relative to unprovoked participants. Experiment 2 replicated and extended Experiment 1 by examining the effectiveness of reappraisal and distraction in the Ultimatum Game. We hypothesized that reappraisal and distraction would both reduce anger quickly. However, because reappraisal involves cognitively processing the anger provocation but distraction does not, we expected anger to flare up in the distraction condition during the bargaining task. For these same reasons, we also hypothesized that participants who reappraised would propose fairer offers and accept more offers than participants in the distraction condition.

## Experiment 1

We conducted Experiment 1 to investigate the effects of intrapersonal anger on economic bargaining behavior. This present study is the first to directly induce anger in economic bargaining by using a relatively strong anger provocation. We investigated the effects of anger on both the proposer and responder roles in the Ultimatum Game. In addition, in contrast to prior research we investigated how participants bargain with a provocateur relative to strangers. Contrasting behaviour toward different bargaining partners may provide insight into whether anger has general or person-specific effects on bargaining outcomes.

Participants gave a brief speech about their life goals to a fictitious participant and were subsequently either insulted or not by the fictitious participant. Next, participants played two economic bargaining games against the fictitious participant and non-provoking control counterparts. This design allowed for separate comparisons of behavior toward the provocateur and non-provoking counterparts. We hypothesized that relative to individuals in the no-provocation condition, provoked participants would experience greater anger, have a more negative impression of the fictitious participant, and punish more during the bargaining games.

### Method

#### Ethics Statement

The project was approved by the University of New South Wales Human Research Ethics Committee (HREC) and all participants provided written informed consent.

#### Participants and Design

A total of 57 students from a public university in Australia participated in exchange for course credit or AUD$10. Seven participants were excluded due to suspicion of the experimental hypotheses or for not following instructions (anger provocation condition *n* = 5; no-provocation condition *n* = 2). A chi-square test for independence with Yates Correction indicated no significant association between experimental condition and suspicion rates, χ^2^ (1, *n* = 57) = .44, *p* = .51, phi = .14. This left 50 participants (30 men; *M_age_* = 20.52 years, *SD* = 4.42; 52% Asian, 36% Caucasian, and 12% other). Participants were randomly assigned to either the anger provocation or no-provocation condition.

#### Materials and Procedure

The study was presented as two unrelated experiments on the effectiveness of computer-mediated negotiation. The first study ostensibly involved piloting a new web conferencing program called “Unilink” that would be used to connect university students across a major Australian city by providing a social support network and facilitating the exchange of ideas. The true purpose of the web conference was to induce anger by delivering provoking feedback. The second part of the study included two bargaining tasks.

Anger Provocation Manipulation: Participants were given 10 min to prepare a 2 min speech based on talking points provided by the experimenter (e.g., life goals), which they would later present via a bogus web conference to a participant ostensibly in the laboratory down the hall. In reality, the web conference was prerecorded. To make this deception more realistic, the experimenter began the web conference with a series of simple instructions for a sex-matched actor, timed to ensure that the instructions given by the experimenter and the responses from the actor were coordinated. The experimenter then instructed the participant and the actor not to interrupt each other during the speeches, which helped ensure that the participant did not discover the deception. The actor always spoke first for 2 min, followed by the actual participant's 2 min speech. Participants were then told that they were to evaluate their partner's speech and vice versa via a single online chat message. In the anger *provocation* condition, participants read that their speech was of poor quality for a university student and that listening to their speech was boring and a waste of time. In the *no-provocation* condition, participants received neutral feedback stating that they had covered and expanded on the main points and discussed each topic separately. This provocation procedure reliably increases anger, aggression, and blood pressure [35], [44], [48].

Bargaining Tasks: Next, participants played two economic bargaining tasks on a desktop computer. To increase the realism of the task, participants were told they were playing for real money to be paid at the end of the experiment by randomly selecting a trial from the bargaining tasks. To lead participants to believe they were playing with multiple players, photographs of all the players including the participant were presented on the monitor [20], [49].

The first bargaining task was based on Kahneman, Knetsch, and Thaler [50], and involved selecting a punishing or non-punishing option. Participants could punish the speech task counterpart by ensuring they received no money, but at a cost to themselves. Specifically, if participants chose to punish, they could allocate $5 to themselves, $0 to the speech task counterpart and $5 to one of the control participants. The non-punishing option was to allocate $6 to themselves, $6 to the speech task opponent, and $0 to the control player. Thus, by choosing to punish the speech task partner, participants chose to retain $1 less rather than allocate money to the speech task partner.

For the second task, participants played a modified version of the Ultimatum Game with the speech task counterpart, and two fictitious counterparts. First, participants played the role of the proposer for a single round by allocating any portion they wished of $10 to the speech task participant and two novel players. Participants always played the proposer first to avoid tit-for-tat retaliation [51]. The order of the players was counterbalanced. Next, participants played the role of the responder in which they received multiple identical offers from the speech task counterpart and two other counterparts combined (half fair – i.e., $5, $4 and half unfair – i.e., $1, $2) [49]. Participants decided whether to accept or reject these offers. On each trial, participants saw a picture and a capital letter identifying the proposer displayed on the screen prior to the offer. Two counterbalanced, pseudo-randomized versions of these offers were used.

Post-Experimental Questionnaire: Participants completed a 28-item Mood Adjective Checklist (MACL; e.g. [52]), nine of which assessed angry affect (e.g., hostile, angry, and annoyed). Participants rated how angry they felt at the beginning of the experiment (α = .90), following the feedback (α = .96), during the Ultimatum Game (α = .95), and at the conclusion of the experiment (α = .96). Participants also rated their counterparts on nine characteristics relevant to economic bargaining (e.g., trustworthiness, fairness; speech counterpart α = .76; other counterparts α = .82). All items were rated on 7-point scales (1 = not at all, 4 = moderately, 7 = extremely). Finally, participants were questioned for suspicion, debriefed, and paid AUD$5 for the Ultimatum Game.

### Results

#### Manipulation Checks

**Figure 1:**
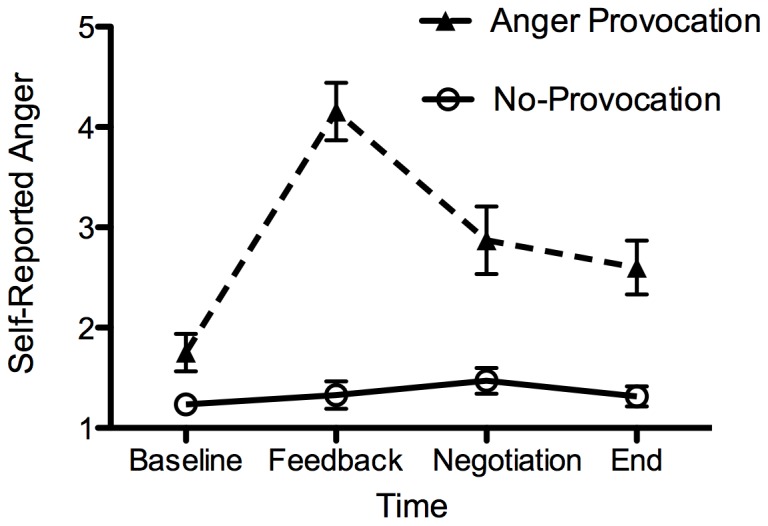
Self-reported anger difference scores as a function of time and provocation condition. Error bars display the standard error of the mean (SEM).

Anger: Participants in the provocation condition (*M* = 1.75, *SD* = 0.93) reported greater anger at baseline relative to the control condition (*M* = 1.24, *SD* = 0.30), *t*(29) = −2.63, *p* = .01, *d* = .74. To control for this difference, we created difference scores by subtracting baseline anger from the three subsequent anger measurements. To investigate whether the feedback conditions differed in anger experienced throughout the experiment, a mixed 3 (time) ×2 (provocation condition) ANOVA using the difference scores was conducted. There was a significant main effect of time, *F*(2,96) = 20.35, *p*<.001, η_p_^2^ = .30, and provocation condition, *F*(1,48) = 17.06, *p*<.001, η_p_^2^ = .26. As hypothesized, these effects were qualified by a significant interaction, *F*(2,96) = 9.22, *p*<.001, η_p_^2^ = .32 (Figure 1). Type I error was controlled with the false discovery rate (FDR), *q*(FDR)<.05. Follow-up comparisons revealed that following the speech feedback, participants in the provocation condition reported a greater increase in anger than the no-provocation condition, *t*(29) = −6.58, *p*<.001, *d* = −1.86. Among those in the provocation condition, these higher levels of anger were sustained during the negotiation, *t*(29) = −2.21, *p* = .04, *d* = −.57, and at the conclusion of the study, *t*(29) = −2.59, *p* = .02, *d* = −.73. These data suggest an effective anger provocation manipulation.

Partner Evaluations: Negative traits were reverse scored and combined into a single variable. We investigated the impression that participants formed of their counterparts using a 2 (provocation condition) ×2 (counterpart) mixed ANOVA. As expected, there was a main effect of provocation, *F*(1,48) = 19.61, *p*<.001, η_p_^2^ = .29, which was qualified by a significant interaction, *F*(1,48) = 10.39, *p* = .002, η_p_^2^ = .18. Specifically, those in the provocation condition (*M* = 3.25, *SD* = .91) rated the speech task counterpart less positively than participants in the no-provocation condition (*M* = 4.41, *SD* = .55), *t*(48) = 5.45, *p*<.001, *d* = 1.54. The no-provocation (*M* = 3.85, *SD* = .64) and provocation conditions (*M* = 3.66, *SD* = .86) did not differ in their impression of the control counterparts *t*(48) = .90, *p* = .37, *d* = .25.

#### Forced Choice Punishment Task

A logistic regression with feedback condition as the independent variable revealed that participants in provocation condition were significantly more likely to choose the punishing option than non-punishing option, *b* = −1.52, *SE* = 0.61, *p* = .01 (percentage punished: 72% provocation, 36% no-provocation). The odds-ratio revealed that participants in the provocation condition were 4.57 times (95% CI = 1.38, 15.11) more likely to select the financially irrational, punishing choice than participants in the no-provocation condition.

#### The Ultimatum Game

**Figure 2:**
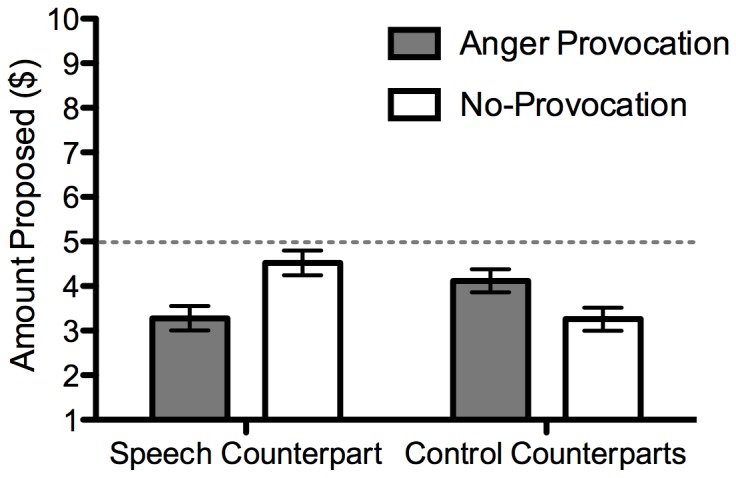
Amount proposed as a function of provocation condition and counterpart type. Error bars display SEM. The dotted line at $5 represents a fair offer.

Amount Proposed: Participants proposed offers to the speech task counterpart and two control counterparts. Proposals to the two control counterparts were averaged as they were highly correlated, *r* = .59, *p*<.001. A 2 (provocation condition) ×2 (counterpart) mixed ANOVA with the latter factor as a within-participants variable, revealed a significant interaction, *F*(1,48) = 18.77, *p*<.001, η_p_^2^ = .28 (Figure 2). Follow-up tests show that as hypothesized, participants in the provocation condition proposed less to the speech task counterpart than those in the no-provocation condition, *t*(46.06) = 3.18, *p* = .003, *d* = .90. Participants in the provocation condition proposed more to the control counterparts than participants in the no-provocation condition, *t*(48) = −2.34, *p* = .02, *d* = −.66. Within-condition comparisons indicated that participants in the provocation condition proposed less to the speech task counterpart relative to the control counterparts, *t*(24) = −2.12, *p* = .05, *d* = −.22. By contrast, those in the no-provocation condition displayed the reverse pattern by proposing more to the speech task counterpart relative to the control counterparts, *t*(24) = 4.52, *p*<.001, *d* = 1.24.

**Figure 3:**
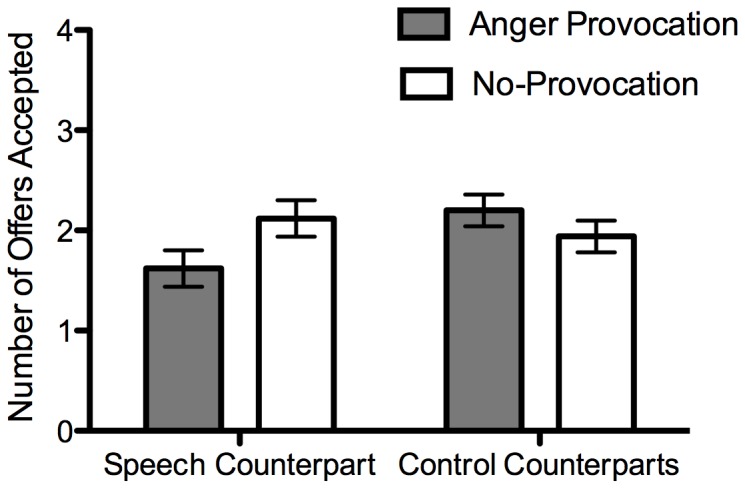
Number of offers accepted as a function of provocation condition and counterpart type. Participants received 8 offers from each counterpart. Error bars display SEM.

Offers Accepted: A 2 (provocation condition) ×2 (counterpart) ×2 (offer fairness) mixed ANOVA with the latter two as within-participants factors revealed a significant interaction between the provocation manipulation and counterpart, *F*(1,48) = 11.08, *p* = .002, η_p_^2^ = .19 (Figure 3). Between group comparisons revealed that participants in the provocation condition were less likely to accept offers from the speech task counterpart than those in the no-provocation condition, *t*(48) = 1.93, *p* = .06, *d* = .55. The provocation condition and no-provocation condition did not differ in the amount accepted from the control counterparts, *t*(48) = −1.17, *p* = .25, *d* = −.33. Within-group comparisons showed that for the provocation condition, participants accepted fewer offers from the speech task counterpart than the control counterparts, *t*(25) = −2.91, *p* = .008, *d* = −.80. Those in the no-provocation condition accepted a similar amount from both the speech task counterpart and control counterparts, *t*(25) = 1.62, *p* = .12, *d* = .46. As expected, there was also a main effect of offer fairness whereby participants accepted more fair offers (*M* = 3.28, *SD* = 1.09) than unfair offers (*M* = .66, *SD* = 1.06), *F*(1,48) = 246.04, *p*<.001, η_p_^2^ = .84. No other effects were significant.

### Discussion

Across two economic bargaining tasks we found that provoked participants punished the speech task counterpart more than unprovoked participants. As hypothesized, angered participants were more likely to give money to a novel participant than the person who provoked them. Angered participants also proposed less fair offers to the speech task counterpart than participants who were not provoked. They were also less willing to accept offers from the speech task counterpart regardless of how fair the offer was. In sum, provoked participants had poorer financial outcomes than unprovoked participants when bargaining with the speech task counterpart. Together these findings suggest that intrapersonal anger adversely influences economic bargaining.

## Experiment 2

One clear implication of Experiment 1 is that effectively regulating anger should improve bargaining outcomes. In Experiment 2, we directly manipulated two emotion regulation strategies. All participants were provoked using the same paradigm as Experiment 1, after which they engaged in 20 min of reappraisal or distraction using a guided writing task. Subsequently participants completed the Ultimatum Game. Relative to the distraction condition, we expected that participants who reappraised would be less angry, propose fairer offers, and accept more offers.

### Method

#### Participants and Design

Participants were 112 students from a public university in Australia who participated in exchange for course credit or AUD$25. Twenty-five participants were excluded from the analyses for suspicion involving the anger induction or for not complying with the instructions (reappraisal, *n* = 11; distraction, *n* = 14). A chi-square test for independence with Yates Correction indicated no significant association between experimental condition and suspicion rates, χ^2^ (1, *n* = 112) = .12, *p* = .72, phi = .05. This left a total of 87 participants: 35 men; *M*_age_ = 21 years, *SD* = 4.96; 51.7% Asian, 32.2% Caucasian, and 14.9% other, 1 unreported). Participants were randomly assigned to the reappraisal (*n* = 44) or distraction condition (*n* = 43). Distraction was chosen as the comparison condition rather than a no-instruction control condition because it is an emotion regulation strategy. Moreover, based on our prior work, when participants were given the opportunity to write about whatever they choose, almost all participants chose to engage in distraction [39].

#### Materials and Procedure

Anger Induction: Participants were told the same cover story as in Experiment 1 stating that they would be piloting Unilink and examining the effectiveness of computer-mediated negotiation. The speech task was identical to Experiment 1. However, all participants received anger-provoking feedback.

Emotion Regulation Manipulation: As reappraisal should occur before an emotion unfolds, participants in the reappraisal condition were given instructions to induce early reappraisal [37]. Specifically, participants in the reappraisal condition were informed prior to the speech task that their partner appeared to be in a bad mood, and not to take it personally. Following the provocation, the emotion regulation task was presented as a study of creative writing. Participants were induced to reappraise or engage in distraction for 20 min by writing about five predetermined topics. Participants in the *reappraisal* condition were instructed to think about the speech task objectively, from the perspective of a third person, and to think about things they learned from the task and found enjoyable (Appendix S1; [37], [53]). For example, “Describe your experience of the Unilink task in a way that makes you adopt a neutral attitude”. Instructions in the distraction condition consisted of emotionally neutral statements (e.g., [40] (e.g., “Write about the layout of the aisles at your local supermarket”).

We ran a third condition in which participants were instructed to ruminate about the provocation (*n* = 45) by writing about their feelings and thoughts they had towards others in the study (e.g., “Write about the feelings you have about the other people you have encountered in the study”). We have successfully used this manipulation in prior research (e.g., [30], [53]–[55]); however, in this experiment, the rumination manipulation check did not reveal a significant effect of emotion regulation condition, *F*(2,129) = 2.40, *p* = .10, *d* = .40. Moreover, participants who ruminated (*M* = 4.33, *SD* = 1.65) used more positive affect words than people in the distraction condition (*M* = 1.66, *SD* = 1.12), *p*<.001, *d* = 1.89, and an equivalent number of positive affect words as people in the reappraisal condition (*M* = 4.54, *SD* = 1.70), *p* = .99, *d* = .13 (which is inconsistent with [44]). This failure to replicate our past research with an identical manipulation suggests that the rumination manipulation was not effective and we therefore focus on the reappraisal and distraction conditions in this paper.

Bargaining Task: Participants completed the modified Ultimatum Game described in Experiment 1.

Post-Experimental Questionnaire: Participants completed four emotion regulation manipulation check items (Appendix S2) assessing the extent that participants reflected on Unilink from an objective and positive perspective during the writing task (α = .55). Next, anger was assessed using the 28-item MACL used in Experiment 1. Anger was retrospectively assessed at the beginning of the study (α = .88), post feedback (α = .91), during the writing task (anger α = .94), during the Ultimatum Game (α = .93) and at the conclusion of the study (α = .94). Next, ratings of all counterparts on nine economic bargaining-relevant characteristics were completed (e.g., trustworthiness, fairness, speech counterpart α = .74; other counterparts α = .67). Finally, participants were probed for suspicion, debriefed, and given AUD$5 for the Ultimatum Game. All items used a 7-point scale (1 = not at all, 4 = moderately, 7 = extremely).

Quantitative Content Analyses: As an additional manipulation check, the writing tasks were analyzed using the Linguistic Inquiry Word Count program (LIWC) [56]. LIWC counts and categorizes words based on a dictionary that contains more than 2,300 words that are grouped in categories [57]. LIWC has been extensively validated [57], [58], and used in research investigating emotion regulation [39], [59]. In our experiment, categories of interest included affect words and causation words. The affect words we examined included anger (e.g., *hate, annoyed, furious*), positive emotion (e.g., *love, nice, sweet*), and negative emotion (e.g., *hurt, ugly, nasty*). Cognitive process words (e.g., *cause, know, ought*) were also investigated, including insight (e.g., *think, know, consider*) and causation words (e.g., *because, effect, hence*).

### Results

#### Manipulation Checks

Self-Reported Emotion Regulation: Participants in the reappraisal condition (*M = *4.06, *SD = *1.02) reported reflecting more on the positive features of the speech task, and thought about it from a more objective perspective than participants in the distraction condition (*M = *2.78, *SD = *1.44), *t*(75.70) = 4.76, *p*<.001, *d* = 1.02, equal variances not assumed and corrected.

Quantitative Content Analyses: To corroborate the self-report data, LIWC was used as a manipulation check. The types of words written in the writing task were analyzed for each emotion regulation condition. As the emotion-regulation conditions varied in the total number of words written, this was used as a covariate for the content analyses (Table 1). Participants in the reappraisal condition used more positive and cognitive mechanism words than participants in the distraction condition.

**Table 1:** Means, standard deviations, and significance tests of words used as a function of linguistic category and emotion regulation strategy.

	Reappraisal		Distraction		Significance Test
	*M*	*SD*	*M*	*SD*	
Word Count	240.34	73.44	273.53	69.44	*F*(1,85) = 4.69, *p* = .03, η^2^ = .05
Anger	0.32	0.57	0.20	0.28	*F*(1,85) = 1.70, *p*>.05, η^2^ = .02
Negative Emotion	1.31	0.96	1.15	0.75	*F*(1,85) = 1.24, *p*>.05, η^2^ = .02
Positive Emotion	4.54	1.7	1.66	1.12	*F*(1,85) = 76.98, *p*<.001, η^2^ = .48
Cognitive Process	18.77	3.68	15.33	2.33	*F*(1,85) = 25.93, *p*<.001, η^2^ = .24

The number of words written was used as a covariate.

Partner Evaluations: Participants rated the speech task counterpart less positively (*M* = 3.16, *SD* = .93) than the control counterparts (*M* = 3.83, *SD* = .72), *F*(1,85) = 35.83, *p*<.001, η^2^ = .30.

#### Self-Reported Anger

**Figure 4:**
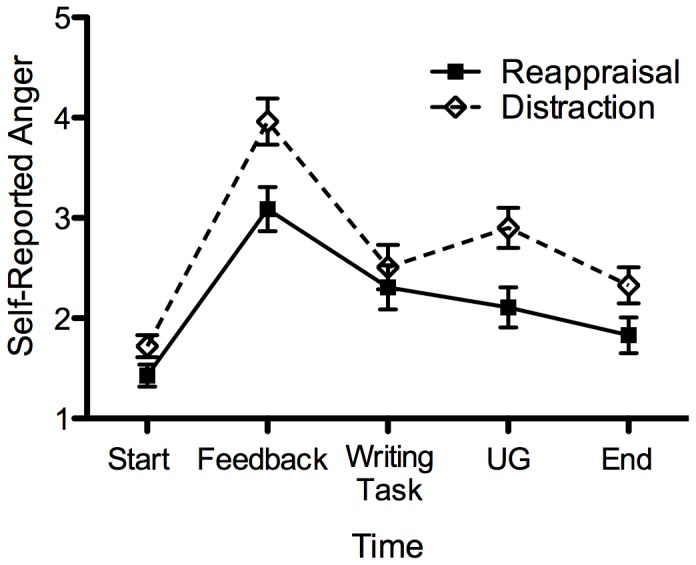
Self-reported anger as a function of emotion regulation condition and time. Error bars display SEM.

A mixed 5 (time) ×2 (emotion regulation condition) ANOVA revealed that there was a main effect of time, *F*(3,292) = 57.36, *p*<.001, η_p_^2^ = .40, Huynh-Feldt corrected, and a main effect of emotion regulation condition, *F*(1,85) = 6.57, *p* = .01, η_p_^2^ = .07. This was qualified by a marginally significant interaction, *F*(3,292) = 2.39, *p* = .06, η_p_^2^ = .03, Huynh-Feldt corrected (Figure 4). We corrected for multiple comparisons using the FDR. There was no difference in anger at the beginning, *t*(66.51) = −1.86, *p*>.05, *d* = −2.11, or end of the experiment, *t*(85) = −1.97, *p*>.05, *d* = 1.95. However, following the provocation, participants in the reappraisal condition reported significantly less anger after the feedback than participants in the distraction condition, *t*(85) = −2.78, *p* = .007, *d* = −2.11. During the writing task, participants in the reappraisal and distraction conditions did not significantly differ in reported anger, *t*(85) = −.67, *p* = .50, *d* = −.61. During the negotiation task, those in the reappraisal condition reported experiencing significantly less anger relative to those in the distraction condition, *t*(85) = −2.75, *p* = .007, *d* = −2.49.

#### Ultimatum Game

Amount Proposed: To examine how much money participants awarded counterparts in the Ultimatum Game, a 2 (emotion regulation condition) ×2 (counterpart) mixed ANOVA was conducted. Contrary to the hypotheses, there was no significant interaction between the emotion regulation condition and counterpart type, *F*(1,85) = 2.59, *p* = .11, η_p_^2^ = .03. However, there was a significant main effect of emotion regulation condition, *F*(1,85) = 5.75, *p* = .02, η_p_^2^ = .06. Participants in the reappraisal condition (*M* =  $3.90, *SD* = $1.25) proposed fairer offers than participants in the distraction condition (*M* = $3.41, *SD* = $1.11). There was also a main effect of counterpart, *F*(1,85) = 32.90, *p*<.001, η_p_^2^ = .28. As expected, participants awarded more to the control partners (*M* = $4.06, *SD* = $1.44) than the speech task counterpart (*M* = $2.85, *SD* = $1.69).

**Figure 5:**
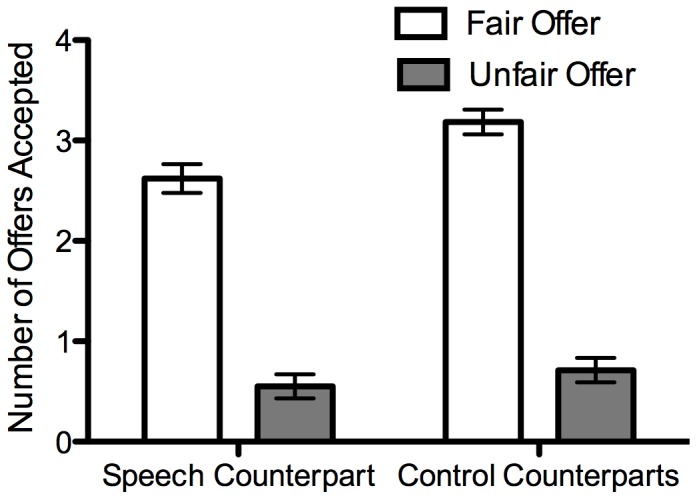
The number of offers accepted as a function of counterpart and fairness of the offer. Participants received 8 offers from each counterpart. Error bars display SEM.

Offers Accepted: A 2 (emotion regulation condition) ×2 (counterpart) ×2 (offer fairness) mixed ANOVA was conducted to examine the number of offers accepted. The main effects of counterpart, *F*(1,85) = 25.25, *p*<.001, η_p_^2^ = .23, and fairness, *F*(1,85) = 277.60, *p*<.001, η_p_^2^ = .77, were qualified by significant interactions. There was a significant counterpart × fairness interaction, *F*(1,85) = 11.08, *p* = .001, η_p_^2^ = .02 (Figure 5). All comparisons were significant, *p*<.05. There was a marginally significant counterpart × emotion regulation interaction, *F*(1,85) = 3.55, *p* = .06, η_p_^2^ = .04; however, follow up comparisons yielded no significant simple effects.

### Discussion

Experiment 2 replicated Experiment 1 as provoked participants proposed and accepted less from the provocateur than the control counterparts. Experiment 2 extended Experiment 1 by examining the effectiveness of two emotion regulation strategies for reducing anger and retaliation. Reappraisal produced the most effective and temporally stable decrease in anger. Distraction was effective in reducing anger immediately following the provocation; however, during the negotiation task, anger increased again. These findings converge with prior research that illustrate the beneficial effects of reappraisal and the short-term effectiveness of distraction for reducing anger [34], [36], [39], [53].

Despite these effects on intrapersonal anger, the influence of emotion regulation strategies on economic bargaining behavior was somewhat more complicated. Reappraisal did not increase fair treatment specifically towards the speech task counterpart. Instead, reappraisal increased the fairness of all offers proposed compared to the distraction condition.

Reappraisal did not affect punitive behavior [20], [23], [43]. When participants were given unfair offers they punished both counterparts equally by rejecting a similar number of offers. However, when the offers were fair, participants rejected more offers from the speech task counterpart than the control counterparts, suggesting retaliation toward the provocateur. In sum, relative to distraction, reappraisal reduced anger during bargaining and improved fair behavior but had no effect on punishment.

## General Discussion

In the present research, we investigated the effects of intrapersonal anger on ultimatum bargaining behavior (Experiment 1) and the relative effectiveness of reappraisal and distraction for reducing anger and retaliation during bargaining (Experiment 2). In Experiment 1, participants who were angered proposed more unfair offers to the provocateur than unprovoked participants. Angered participants accepted fewer offers from the provoking counterpart than the control counterpart despite receiving the same offers from both parties. The results of Experiment 2 partially supported the hypothesis that reappraisal would improve bargaining outcomes. Specifically, participants who reappraised proposed fairer offers to all counterparts relative to the distraction condition, suggesting that reappraisal increased prosocial bargaining behavior. However, reappraisal had only weak, non-significant effects on increasing the number of offers accepted.

Together, the results of Experiments 1 and 2 are broadly consistent with past research on the intrapersonal effects of anger in economic bargaining [13]. We found angry participants were less likely to reward the anger-provoking counterpart. Moreover, we extended previous research by examining the role of the proposer and by using an interpersonal provocation to investigate negotiating with a disliked person.

In accordance with the process model of emotion regulation, reappraisal should be most effective when introduced prior to a full-blown anger response [37]. Doing so changes the meaning of the provocation. As a consequence of reappraising, the hostile social interaction had reduced emotional impact and anger remained low during bargaining. By contrast, the process of distraction directs attention away from the source of anger without reinterpretation. Thus, distraction should only provide temporary relief, as it does not resolve the underlying cause of anger. Consequently, once participants in the distraction condition encountered the provoking counterpart again in the Ultimatum Game, their self-reported anger increased. Seeing the anger-provoking person again served as a reminder of the original provocation, which likely reactivated anger-related associative networks [60], [61]. These findings are consistent with the notion that reappraisal entails reinterpreting the anger-inducing event in a way that reduces the emotional impact. In the longer term, bargaining may occur several hours or days after the initial provocation. Yet it is not known whether reappraisal initiated later in time, may still be effective for reducing anger and retaliation in economic bargaining. Prior research has suggested that late reappraisal may be quite effortful [62], [63]. Further research is required to examine the longer-term effects of reappraisal and distraction in economic bargaining.

One key contribution this article makes to the existing literature is by differentiating between the effects of reappraisal and distraction in economic bargaining. Contrary to past research, we did not find that distraction and reappraisal have equivalent effects on economic bargaining [43]. Instead, we found that participants who reappraised made fairer offers than participants who distracted themselves, suggesting that participants who reappraised were more likely to overcome their feelings of anger and therefore award fairer treatment to the provocateur.

There are several methodological and theoretical reasons to explain the divergent findings between Wang and colleagues and the present study [43]. First, we used a stronger anger provocation, as participants disclosed personal information prior to receiving the provocation. Second, we examined the longer-term effectiveness of reappraisal and distraction by using a longer bargaining task that included multiple offers and a 20 min regulation period. This is in contrast to the single $2 offer and 30 sec to 2.5 sec delays used by Wang and colleagues. Taken together, these studies complement each other. These findings suggest that distraction may be a useful strategy in brief one-shot instances when confronted with a mild provocation, such as proposing an unfair offer. However, in extended, more personally relevant situations with a stronger provocation, distraction may be less effective for regulating affect and behavior.

Our data also speak to the adverse effect of anger on social relationships during negotiation. In addition to considering the objective fairness of the offer, participants also based their decision on who proposed the offer. Provoked participants were less economically rational when playing with the provoking person and more objective when playing with strangers. This suggests that the emotions participants experienced and the intentions participants may have attributed to their counterpart impacted behavior in the Ultimatum Game. Moreover, participants reported disliking the anger-provoking counterpart more than the control counterparts. This converges with Sanfey et al. [20] who found that participants were more likely to accept unfair offers from computers than humans.

On the bright side, our data also highlight the positive aspects of self-disclosure in bargaining contexts [64], [65]. Specifically, in Experiment 1, after giving and listening to a brief speech about personal topics, participants who were not provoked proposed fairer offers to the person they self-disclosed with during the speech task relative to strangers. This favorable treatment, however, did not extend to the responder phase of the Ultimatum Game. Given the large number of offers, participants may have applied a decision rule to not accept unfair offers. Furthermore, participants may have become disheartened when they received unfair treatment in the Ultimatum Game, as half the offers were unfair.

The present experiments focused on the intrapersonal effects of anger. By contrast, interpersonal anger can serve as a powerful signal in bargaining [1], [66]. For example, in the Ultimatum Game, a responder who has a reputation for being easily angered may elicit a fairer offer from the proposer. As a consequence, the proposer chooses not to act in their own self-interests by proposing the lowest possible offer. In this way, emotions may serve as commitment devices that can elicit cooperation and encourage individuals to sacrifice short-term self-interests for longer-term concerns [67]. Indeed, in a modified version of the Ultimatum Game, participants made more fair offers to angry opponents than happy opponents [68].

There were some limitations associated with the present study. First, to reduce suspicion, affect was measured at the conclusion of the study. This technique avoids increasing awareness of one's emotional state and possible mood-repairing action. However, it can also induce retrospective biases in recall. Second, the traditional Ultimatum Game has strong fairness norms that may have reduced the effect of emotion regulation on behavior. Future research could investigate whether tasks that require greater elaboration of thought rather than quick decision-making across multiple trials may be more amenable to the positive effects of emotion regulation.

The Ultimatum Game has been frequently used in past research [13], [20], [49] and has parallels with real life bargaining including final ultimatum offers in bargaining. The online bargaining format used is also present in some real life bargaining situations including eBay auctions. However, the Ultimatum Game is an artificial model of bargaining, which limits the external validity of the results. For instance, in the present research there was no broader context or interaction between bargaining parties, and participants were limited to a set number of response options. To address this issue, future research could investigate the extent to which anger reappraisal may benefit or jeopardize real world bargaining.

Future research could further explore when to use reappraisal and distraction, how to implement reappraisal, and why reappraisal reduces anger in bargaining. For example, as reappraisal had longer lasting effects on lowering anger than distraction, reappraisal may be more appropriate for reducing anger in longer-term repeat bargaining. Distraction may be more effective for reducing anger in instances where reappraisal is not possible or may be detrimental. Further research is also required to determine the timing necessary for reappraisal and distraction to exert optimal effects on anger and bargaining behavior. We investigated early reappraisal supplemented by a later more detailed induction. Others have shown that late distraction is more effective than late reappraisal for reducing sad affect [69]. If people are not prepared for being confronted with a hostile bargaining partner, reappraisal may not be more effective than distraction.

Further research is also necessary to identify the mechanisms underlying how reappraisal influences anger and bargaining behavior. For instance, reappraisal may reduce the amount of anger experienced, reduce the likelihood that people will act on their anger, or may involve both functions. Our findings suggest that the combination of both an antecedent-focused reappraisal induction and a reappraisal writing task reduced self-reported anger and increased prosocial bargaining behavior. Future research could orthogonally manipulate early versus late reappraisal to determine which is most effective in bargaining contexts.

### Conclusion

This research demonstrated that intrapersonal anger can adversely impact bargaining. The current findings suggest that reappraisal may be more effective than distraction for regulating anger in an ultimatum bargaining context. The behavioral findings also suggest that reappraisal resulted in fairer treatment when making offers but did not extend to accepting and rejecting offers. In terms of concrete implications for bargainers, reappraisal is likely to be more adaptive than distraction for regulating affect and retaliatory behavior when the economic bargaining is personal, relatively long in duration, and when repeat bargaining or future contact is likely to occur. Although more work remains to be done, our results provide initial evidence that effective reappraisal may improve financial outcomes and decrease anger in bargaining.

## Supporting Information

Appendix S1 **Emotion Regulation Writing Task.**
(DOCX)

Appendix S2 **Reappraisal Manipulation Check.**
(DOCX)
