# Supplementary material for: The Effects of Intrapersonal Anger and Its Regulation in Economic Bargaining
Source: PLoS One. 2012 Dec 26;7(12):e51595. doi: 10.1371/journal.pone.0051595 (PMC3530551; doi:10.1371/journal.pone.0051595)
Supplement: Appendix S1 — Emotion Regulation Writing Task. (DOCX) [file pone.0051595.s001.docx]

**Appendix S1. Emotion Regulation Writing Task**

**Reappraisal**

Write about the Unilink task from the perspective of an objective, third person observer.

Describe the people that you have encountered during the Unilink task objectively and

analytically.

Describe your experience of the Unilink task in a way that makes you adopt a neutral attitude.

Describe any positive things that you might have learnt from the Unilink task.

Describe at least one interesting or enjoyable feature of the Unilink task.

**Distraction**

Describe the inside and outside of the Mathews building.

Write about pigeons pecking on the ground at a local park.

Write about the layout of the aisles at your local supermarket.

Describe the layout and contents of a typical classroom.

Write about how a ball point pen works.
