# Supplementary material for: The Effects of Intrapersonal Anger and Its Regulation in Economic Bargaining
Source: PLoS One. 2012 Dec 26;7(12):e51595. doi: 10.1371/journal.pone.0051595 (PMC3530551; doi:10.1371/journal.pone.0051595)
Supplement: Appendix S2 — Reappraisal Manipulation Check. (DOCX) [file pone.0051595.s002.docx]

**Appendix S2. Reappraisal Manipulation Check**

During the writing task, to what extent did you reflect on positive features of the Unilink task?

During the writing task, to what extent did you think of the Unilink task from an objective perspective?
